# Improving the outcome of infants born at <30 weeks' gestation - a randomized controlled trial of preventative care at home

**DOI:** 10.1186/1471-2431-9-73

**Published:** 2009-12-03

**Authors:** Alicia J Spittle, Carmel Ferretti, Peter J Anderson, Jane Orton, Abbey Eeles, Lisa Bates, Roslyn N Boyd, Terrie E Inder, Lex W Doyle

**Affiliations:** 1 Physiotherapy, School of Health Sciences, University of Melbourne, 200 Berkley Street, Parkville, Victoria, 3052, Australia; 2 Victorian Infant Brain Studies, Murdoch Childrens Research Institute, 2ndFloor, Flemington Road, Parkville, Victoria, 3052, Australia; 3 Neonatal Services, Royal Women's Hospital, 7th Floor, Cnr Grattan Street and Flemington Road, Parkville, Victoria, 3052, Australia; 4 Psychology, School of Behavioural Sciences, University of Melbourne, 12th Floor, Redmond Barry Building, Victoria, 3010, Australia; 5 Department of Obstetrics and Gynaecology, 7th Floor, Cnr Grattan Street and Flemington Road, Parkville, Victoria, 3052, Australia; 6 Queensland Cerebral Palsy and Rehabilitation Research Centre, School of Medicine, University of Queensland, Level 3, Foundation Building, Royal Children's Hospital, Herston, Queensland, 4006, Australia; 7 Newborn Medicine, Washington University School of Medicine, 660 S. Euclid Avenue, St. Louis, MO, USA

## Abstract

**Background:**

Early developmental interventions to prevent the high rate of neurodevelopmental problems in very preterm children, including cognitive, motor and behavioral impairments, are urgently needed. These interventions should be multi-faceted and include modules for caregivers given their high rates of mental health problems.

**Methods/Design:**

We have designed a randomized controlled trial to assess the effectiveness of a preventative care program delivered at home over the first 12 months of life for infants born very preterm (<30 weeks of gestational age) and their families, compared with standard medical follow-up. The aim of the program, delivered over nine sessions by a team comprising a physiotherapist and psychologist, is to improve infant development (cognitive, motor and language), behavioral regulation, caregiver-child interactions and caregiver mental health at 24 months' corrected age. The infants will be stratified by severity of brain white matter injury (assessed by magnetic resonance imaging) at term equivalent age, and then randomized. At 12 months' corrected age interim outcome measures will include motor development assessed using the Alberta Infant Motor Scale and the Neurological Sensory Motor Developmental Assessment. Caregivers will also complete a questionnaire at this time to obtain information on behavior, parenting, caregiver mental health, and social support. The primary outcomes are at 24 months' corrected age and include cognitive, motor and language development assessed with the Bayley Scales of Infant and Toddler Development (Bayley-III). Secondary outcomes at 24 months include caregiver-child interaction measured using an observational task, and infant behavior, parenting, caregiver mental health and social support measured via standardized parental questionnaires.

**Discussion:**

This paper presents the background, study design and protocol for a randomized controlled trial in very preterm infants utilizing a preventative care program in the first year after discharge home designed to improve cognitive, motor and behavioral outcomes of very preterm children and caregiver mental health at two-years' corrected age.

**Clinical Trial Registration Number:**

ACTRN12605000492651

## Background

With increasing numbers of very low birthweight (VLBW; <1500 g) or very preterm (<30 weeks' gestational age) infants surviving, there is a growing recognition that substantial numbers of these infants will develop motor, cognitive and behavioral problems. These disabilities have life long consequences and will soon translate into increased numbers of adults with disabilities[1]. The educational and social implications are substantial. The prevention of learning disabilities, behavior and motor problems is an important goal for modern perinatal care for preterm children and their families[2].

There are many potential factors contributing to the risk of disability in the preterm infant, including severity of illness, suboptimal nutrition, therapeutic exposures, environmental and social factors. Whilst there are many randomized controlled trials investigating medical interventions that target some of these areas, there is a paucity of sufficiently powered randomized trials involving multi-dimensional interventions focusing on environmental, behavioral, and early developmental factors [3].

This study, called VIBeS (Victorian Infant Brain Study) Plus, follows on from the earlier VIBeS study that monitored the natural history of brain development by evaluating alterations in brain structure in the early weeks after birth with advanced neuro-imaging modalities, and compared brain development, motor and behavioral outcomes at 2 years[4,5]. The current VIBeS Plus study involves a randomized controlled trial of a preventative care program with the aim to improve the infant's cognitive and motor development, behavioral regulation, as well as the primary caregiver-child interactions and primary caregiver mental health at 24 months' corrected age.

The VIBeS Plus program is partly based on a study by Dolby et al[6], with the content of this intervention package updated to incorporate several related recent theoretical frameworks of development, including dynamic systems theory[7] and family-centered care[8]. The program by Dolby et al[6] involved two teams, each of a psychologist working collaboratively with a physiotherapist to develop a strong link between behavioral regulation and motor competence. The study by Dolby et al was a quasi-randomized trial with three groups with 81 subjects entered (27 in each group) with 69 subjects completing the study in the preterm intervention group (n = 24), a preterm control group (n = 22) and a term control group (n = 23).

The program by Dolby et al[6] consisted of six sessions, with the family first seen in hospital at 36 weeks' postmenstrual age. The following sessions occurred at home: at term, 1, 4, 6 and 9 months' corrected age. Assessments and interventions were targeted to occur at the beginning of a new developmental phase, where there is often a period of disorganization prior to change[9]. At 12 months, infants in both groups were assessed by a psychologist who was masked to the infant's status (preterm vs term, and intervention vs control) using the Bayley Scales of Infant Development (BSID)[10]. The authors reported that there were significant effects in favour of the intervention group for both cognitive and motor outcomes[11].

The program by Dolby et al[6] was designed specifically for parents when their infants were at home, and was based upon the framework of the synactive model of self regulation and developmental care described by Als[12,13] and attachment theory described by Sroufe[14]. Each model described in the study of Dolby et al[6] is outlined briefly below.

### Self-regulation model

Preterm infants have been reported in many studies to have alterations in behavioral regulation compared with infants born at term[15]. These alterations in behavioral regulation may lead to a preterm infant being more irritable, taking longer to settle into a routine, and being less playful[16,17]. Als[12] proposed a synactive model of neonatal behavioral organization to help explain these alterations in neurobehavior and a framework for intervention. The underlying assumptions of the model are that five systems, including motor, state organization, autonomic, attention-interaction and self regulation systems, work continuously in an inter-relationship for the infant to function[12,18]. Any alterations within the five systems will cause functional difficulties for the infant and affect overall self regulation. Caregivers are encouraged to enhance the self regulation abilities of the individual baby by supporting any of the five systems, such as supporting motor regulation by containing the infant with swaddling. The majority of research on interventions using this model have focused on the infant in the hospital environment, with reports of varying success[19,20]. Dolby et al[6] designed their study to incorporate the principles of self-regulation but hypothesized that a home-based program would be more beneficial as it could target the parents specifically.

### Attachment Theory

Attachment refers to the relationship and special emotional bond that infants develop with their caregivers over the first year of life[21]. Attachment is thought to develop over a period of time, as the adult and infant engage in interaction, and the infant organises his or her behavior around the care givers[21]. Attachment theory is based upon the biological need an infant has to interact with their environment[22]. From birth, infants can attend to the sound of a voice, and be soothed by human touch[23]. As mentioned previously, preterm infants may have altered self-regulation which can affect their ability to interact with their environment, and caregiver responsiveness is required. Positive early interactive experiences with a primary caregiver are believed to be an important process in "fine-tuning" the neural developmental systems and creating a secure infant. Attachment behavior and organization evolves in phases and is dependent on the age of the child[21]. During the first phase, from birth to three months, the caregiver learns to interpret and respond appropriately to the infant's fluctuating states and behavioral cues. The caregiver has an important role in setting limits of stimulation so the infant can remain organized. During the second phase, from three to six months, the infant becomes more communicative and responsive to their caregivers. The caregiver engages, relaxes, then re-engages with the infant in response to the infant's signals. Consequently, the infant can learn to maintain organized behavior with increasing levels of stimulation[22]. The infant may initiate engagement, but cannot maintain organization independently, and the caregiver thus has an important role in controlling their infant's state and behavior. During the next phase, from 6-12 months, the infant becomes more mobile and has an increased capacity for intentional behavior with the development of more motor control. A reciprocal attachment relationship develops when there has been a history of appropriate caregiver-orchestrated interactions during the first six months. The caregiver has an important role in providing a secure base for their infant so the infant can learn to be independent whilst being able to check that the caregiver is present, if needed [9].

Parents of infants born preterm may experience a range of responses following the birth, including depression [24-27], anxiety [28-30] and symptoms of post traumatic stress disorder[31]. Studies have also shown that parental stress is related to less sensitive, more intrusive and more active maternal interactional behavior[32]. There is some evidence that programs that are aimed at improving the parent-child interaction through providing emotional support in the way of information and education of infants' behavioral cues, encouragement and empathy increased parental feelings of self confidence and competence, and increased positive parent-infant interaction [33-35]. The study by Dolby et al[6] used the three phases of attachment development described by Sroufe above [22] to guide their intervention program.

Whilst the program by Dolby et al[6] has a strong theoretical background, the methodology (study design and subject numbers) and reporting of the results limits the conclusions that can be drawn from the study. Therefore, we conducted a randomized controlled trial, partly based upon the theoretical content of the Dolby et al[6] study, with the aim of improving the infant's motor and cognitive performance, the parent-infant relationship, and primary caregiver mental health outcomes. The original intervention model reported by Dolby et al[6] which was created in the 1980s, has been updated in the present intervention to account for current theories of development and research on early intervention[3]. In addition, this intervention targets very preterm infants who have been identified as being at high risk for developmental impairment. The VIBeS Plus program was designed for infants born at younger gestational ages (all infants will be born at <30 weeks' gestational age whereas the mean gestational age of the Dolby study infants was 32 weeks), with a higher incidence of multiple births, and with the study commencing later, at 40 weeks' gestation rather than before 40 weeks in the NICU. We have published a Cochrane review of early developmental interventions that demonstrated that interventions commencing post hospital discharge had similar effects on cognitive and motor development compared with interventions that commenced in the NICU[3]. The theoretical framework for the VIBeS Plus program incorporates the theoretical models used in the study by Dolby et al[6] with the inclusion of dynamic systems theory and family centered practice model.

### Dynamic Systems Theory

Dynamic systems theory is a conceptual framework that evaluates motor behavior as emerging from the dynamic cooperation of many subsystems in a task specific context[7]. Dynamic systems are any system that changes over time, including not only the infant's central nervous system but also the biomechanical, psychological and social environments. This contrasts with the traditional beliefs about motor development, whereby the infant's motor performance is dependent on the interactions between the infant's inherent and emerging skills with that of the environment in which the task is being performed and the characteristics of the desired task[36].

Development is considered to be non-linear within this model, such that the infant may have times of rapid development and times where the infant is refining their skills. Infants will experience 'transition phases' associated with the infant learning a higher level skill[7]. During this time the infant's movement becomes highly variable as the infant experiments with different movement patterns. As the infant learns the most efficient way of moving, the skill will be refined. This model is consistent with Als' synactive model of neonatal behavioral organization[12] and Brazelton's theory of touchpoints[9]. Integration of new motor strategies occurs through a process of neuronal selection, in which neuronal connections associated with the most efficient movement patterns are strengthened through repeated use[37]. By creating an enriching environment, the infant should be motivated to move to interact with their surrounds. Our intervention is timed at stages of transition so the therapist has the ability to view how the infant learns and adapts to new tasks[38].

### (a) Family-centered care

The intervention program involves "family-centered care", where the health professional recognises the important role that families play in ensuring the health and well-being of children[39]. Intervention programs have typically been child-centered, with therapists being viewed as the "expert" and setting goals that focus on bringing about changes in the child[8]. However, this form of intervention is being challenged by family-centered practice which views the parent as knowing their child best, being a valuable resource and having tremendous insight into their child's abilities. Parents are increasingly involved in implementing home programs not only because of resource limitations within the healthcare system, but also because of the importance of family-centered care[40]. The principles of family-centered care are that the health professional recognises each child and their family's innate strengths, with the health care experience being viewed as an opportunity to build on strengths, and to support families in the care-giving and decision making roles. It identifies that each family is different and that the children function optimally within a supportive family and community context[8]. Family involvement is a key to intervention, as it means that activities can be incorporated into daily life and the infant can learn through repeated reinforcement of an activity. For example, if the therapist and family are working together on encouraging flexed, contained postures to help the infant settle, the family can hold the infant in flexion during feeding, nappy changes, play etc. The relationship between family and professionals is a partnership in which the families define the priorities for therapeutic intervention, with the therapist helping to direct the intervention process.

### VIBeS PLUS

The VIBeS Plus program is home-based with the infant's primary caregiver a central component in the intervention. Intervention commences early in the developmental process, at term, so that various subsystems that influence development from the musculo-skeletal system to the parent-infant relationship can be enhanced within weeks of the baby being discharged home. At each stage components of the intervention will take account of the infant's development, caregiver-infant interaction and the environment. The program employs a problem-based learning approach where the caregiver's concerns are addressed and strategies devised together with the therapist, rather than the didactic approach where an expert delivers therapy or teaches the mother.

## Methods/Design

The aim of this randomized controlled trial is to compare the VIBeS program, which is delivered over nine sessions by a team of a physiotherapist and psychologist, with routine post-discharge care. The outcomes for this trial are the infant's cognitive, motor, and language development, and behavioral regulation, as well as caregiver-child interaction and caregiver mental health at 24 months of age, corrected for prematurity. The study has been approved by the Royal Women's Hospital and Royal Children's Hospital Research and Ethics Committees.

### Study sample

Infants born at <30 weeks' gestational age with no major congenital anomalies associated with a poor neurodevelopmental outcome will be eligible for inclusion in this study. As the study involves home-based assessment and intervention, families will be required to live within a 100 km radius of the hospital. Families will need to speak English, as funding is not available for interpreters. Infants will be excluded if they are still in hospital at 4 weeks' corrected age, as the study is designed to target the primary care giver and infant in a home-based setting. Infants will be recruited from the Royal Women's Hospital or the Royal Children's Hospital, Melbourne, Australia. These hospitals represent two of the four neonatal intensive care units in the state of Victoria, Australia.

### Sample Size Calculations

The primary basis for sample size calculation is the comparison between treatment and control groups on cognitive and motor outcomes at 24 months of corrected age. We consider an important clinical difference to detect would be an improvement of at least 0.4 SD (6 points) in the cognitive and/or the motor scales of the Bayley Scales of Infant and Toddler Development - 3^rd ^edition[41]. With a type-I (alpha) level of 0.05, and 80% power, we will require 100 subjects in each group, i.e., a sample size of 200.

### Recruitment procedure

Infants will be enrolled at 38-40 weeks, after parental consent is obtained. All eligible infant's mothers, fathers and/or primary caregivers will be approached by a research nurse who is responsible for all recruitment, 4-8 weeks after birth when survival of the infant beyond the primary hospitalization period seems likely. Families will be given a parent information statement regarding the purpose of the program and consent forms to participate in the study.

### Study time line and protocol

**Figure 1:**
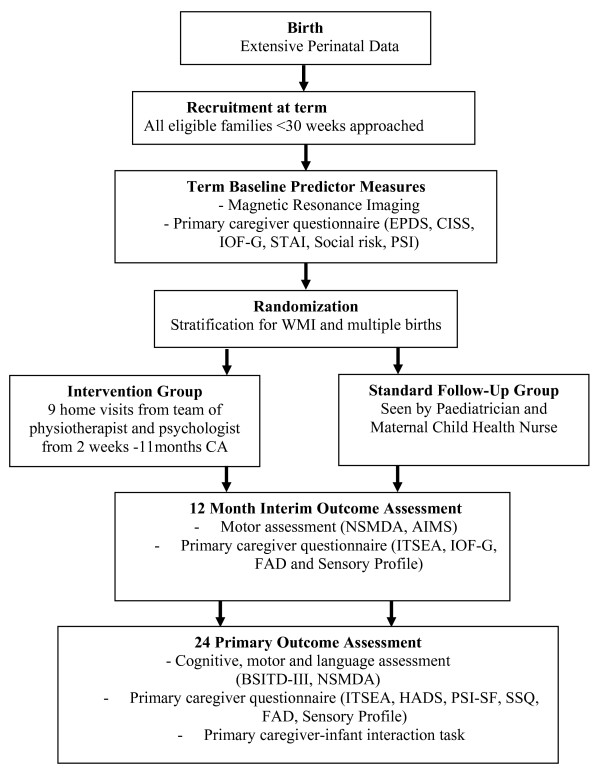
**Study time line**. - EPDS = Edinburgh Postnatal Depression Scale[44]; CISS = Coping Inventory for Stressful Situations[46]; IOF-G = Impact of Family Scale[47]; STAI = State Trait Anxiety Inventory; Social Risk= Social risk Index[41].; PSI= Parenting Stress Index[45]; WMI = white matter injury[50,51]; BSITD-III = Bayley Scales of Infant and Toddler Development -3^rd ^edition[40]; AIMS = Alberta Infant Motor Scale [57]; NSMDA=Neurological Sensory Motor Developmental Assessment[56]; ITSEA = Infant Toddler Social Emotional Assessment[58]; HADS = Hospital Anxiety and Depression Scale[59]; PSI-SF = Parenting Stress Index - Short Form[61]; SSQ = Social Support Questionnaire[63]; FAD = Family Assessment Device; Sensory profile = Infant Toddler Sensory Profile Questionnaire [67].

At 38-42 weeks' postmenstrual age following consent to participate, all families will complete baseline questionnaires and perinatal data will be collected by the research nurse. Infants whose families consent to magnetic resonance imaging (MRI) will be scanned at term equivalent age. Following the MRI, or at term equivalent age if MRI is not performed, infants will be randomized to intervention or standard follow-up groups. Infants and families in the intervention program will have nine home visits provided by a team of a psychologist and a physiotherapist throughout the first year. All infants in the intervention and standard follow-up groups will be seen at 12 months' corrected age for motor assessments and parents will be asked to complete questionnaires. At 24 months' corrected age all children will participate in cognitive and motor assessments, along with a primary caregiver-infant interaction task. In addition, the primary caregiver will complete questionnaires at this time (Figure 1).

### Perinatal data collection

Perinatal data including information on the pregnancy, birth history, and neonatal course (e.g. gestational age, birthweight, gender, multiple birth status, cranial ultrasound findings, proven or suspected necrotizing enterocolitis, maternal antenatal corticosteroid administration, postnatal corticosteroid use and use of oxygen at discharge from hospital) will be collected by the research nurse from medical files and the hospital neonatal database. The primary caregiver will be asked to complete questionnaires when their infant is at term equivalent age, prior to randomization, to obtain the following information:

- *Social Risk *[42]: will be assessed using a 12-point index comprising of six aspects of social status including family structure, education of primary caregiver, occupation of primary income earner, employment status of primary income earner, language spoken at home, and maternal age at birth. Based on the overall risk score, each family will be categorized as lower social risk (0-1) or higher social risk (2+).

- *Edinburgh Postnatal Depression Scale (EPDS) *[43]: will be used to assess primary caregiver depressive symptoms at baseline. It is a widely used 10 item screening tool for postpartum depression. Each item contains a statement such as "I have felt sad or miserable" with four possible responses ranging from "No, not at all" to "Yes, most the time." The primary caregiver is directed to "Choose the answer that comes closest to how you have felt in the past seven days." Each item is scored from 0 to 3 in terms of severity, giving a total score ranging from 0 to 30. The EPDS has demonstrated high reliability and specificity as an indicator of significant depressive symptomatology in new mothers[44]. In this study a cut-off of 12 will be used to indicate depressive symptomatology based upon validation studies[44,45].

- *The Life Stress scale from the Parenting Stress Index *[46]: will be used to examine potentially stressful events within the last 12 months, and whether the respondent thinks that they have a continuing impact. The Life Stress scale is a 19 item scale assessing the number of stressful situational circumstances often beyond the control of a parent (eg. death of a relative, loss of a job). It provides an index of the amount of stress outside the parent-child relationship that the parent is currently experiencing.

- *The Coping Inventory for Stressful Situations (CISS) *[47]: which is a 48-item inventory will be used to measure three major types of coping styles in an individual, including Task-Oriented (problem-solving), Emotion-Oriented (focuses on consequent emotions, becoming angry/upset), and Avoidance Coping (Distraction and social diversion). Primary caregivers will be asked to rate each item on a five point scale ranging from (1) "not at all" to (5) "very much".

- *Impact on Family Scale (IOF-G) *[48]: will be used to assess the effect of a child's illness on the family system in relation to economic, social, familial, and overall strain. There are 24 items scored on a 4 point Likert scale - (strongly agree to strongly disagree) which measure 4 dimensions of impact upon the family: Financial (economic consequences for the family), Familial/Social (disruption of social interaction), Personal Strain (psychological burden experienced by the primary caregiver and Mastery (coping strategies by the family).

- *The State-Trait Anxiety Inventory (STAI) *[49]: will be used to differentiate between anxiety symptoms with regard to both a current, temporary 'state' of anxiety and a more long standing personality quality 'trait'. It is a 40-item self-report scale divided into two sections, each having 20 questions. Each statement contains a 4 point Likert scale (1= not at all, 4= very much so). Higher scores indicate higher levels of anxiety. For the current study, we will utilized state anxiety to evaluate parents' current anxiety, rather than more stable personality attributes.

### Magnetic Resonance Imaging (MRI)

#### Procedure for MRI

Brain MRI will be performed at 38-44 weeks' postmenstrual age at the Royal Children's Hospital. Infants are still eligible for the trial if parents do not consent to MRI. For the MRI, infants will be fed, fitted with earmuffs to minimise noise exposure, then carefully wrapped and placed in a vacuum fixation beanbag (S&S Radiographic Products, Brooklyn, New York) designed to keep the infant still and supported in the scanner[4] All imaging will be obtained without sedation or anaesthesia. Infants will be closely monitored during the MRI scan by electrocardiography and pulse oximetry. MRI will initially be performed using a 1.5 Tesla General Electric Sigma System (General Electrics-Medical Systems, Milwaukee, Wisconsin). When the scanner is upgraded, infants will be scanned using a 3.0 Tesla Siemens Trio (software version 11b and 13b) using a standard 12 channel matrix head coil operating in CP mode.

### MRI Protocols

Brain development at baseline will be assessed using advanced MRI techniques to qualitatively assess cerebral structure. For the 1.5 Tesla scanner two different imaging modules will be applied to the acquisition of the primary MR data, a T1 3D Fourier transform spoiled gradient recalled sequence (1.5 mm coronal slices; flip angle 45°; repetition time (TR) 35 msec; echo time (TE) 9 msec; field of view 18 cms; matrix 256 × 256) and a dual-echo proton density and T2 weighted spin echo sequence (1.6 mm coronal or 3 mm axial slices; flip angle, 90E; repetition time 4000 msec; echo time, 70 and 140 msec; field of view, 18 cm; matrix 512 × 512 were used). These sequences provide images with minimal noise and intensity artefacts. For the 3.0 Tesla 76 contiguous Coronal Dual Echo Turbo Spin Echo T2 weighted images (1 × 1 × 2 mm) will be acquired with TR = 4900 ms, TE = 64/179 ms and a parallel imaging factor of 2 (GRAPPA algorithm) with refocusing flip angle 150°. T1 weighted imaging was performed using 176 contiguous slices with FLASH 3D acquisition (1 × 1 × 1 mm) TR = 19 ms, TE = 4.92 ms, Flip 25° and a parallel imaging factor of 2 (GRAPPA algorithm).

#### MRI Qualitative Scoring

A standardized qualitative structural scoring system will be used to assess white and grey matter abnormalities[50,51]. All scans will be scored independently by a pediatric neuroradiologist or neonatologist without prior knowledge of clinical status. This method has been reported to have excellent predictive validity and reliability[52]. White matter abnormality (WMA) will be graded using five items including: 1) the nature and extent of white matter signal abnormality; 2) periventricular white matter volume loss; 3) the presence of any cystic abnormalities; 4) ventricular dilatation; and 5) thinning of the corpus callosum. Grey matter injury will be graded using three items assessing: 1) the presence of grey matter signal abnormality; 2) the quality of gyral maturation; and 3) the size of the subarachnoid space. White matter abnormality will then be further classified by the composite scores of these five categories (potential range in scores 5-15) to: no injury (score 5-6); mild injury (score 7-9); moderate injury (score 10-12) or severe injury (score 13-15)[4]. Grey matter will be categorized as normal (score 3-5) or abnormal (score 6-9).

### Randomization Process

Infants will be randomly allocated to the intervention and control groups by a computer-generated random sequence, with the treatment allocation concealed in opaque envelopes; only the trial statistician will have access to the code. As parents have the choice to participate in the study without their infant undergoing brain MRI, there will be three strata: 1) nil to mild white matter abnormality, 2) moderate to severe white matter abnormality, or 3) no-MRI group. Secondly, as infants of multiple births need to be randomized to the same group due the family being involved in the intervention, there will also be stratification for multiple births.

### Intervention versus control group

Parents will be notified of group allocation following completion of baseline questionnaires and the randomization process. The two groups are "control" or "intervention".

#### Control group

There are no standardized protocols for medical follow-up at the Royal Women's Hospital and Royal Children's Hospital, particularly as the majority of very preterm infants are discharged from these hospitals to another hospital before going home, and follow-up then occurs at the step-down hospital. Each child has access to a maternal child health nurse in the community. Referral can be made to early intervention services by the infant's health care team at any time during the intervention. A record sheet for visits to doctors, nurses and other health professionals will be given to families.

#### Intervention group

The intervention group will also receive standard medical and nursing follow-up, but, in addition, will receive the preventative care program from 1-2 weeks' post-term age until 11 months of corrected age. The preventative care program is described in the following section.

### VIBeS Plus Intervention

The intervention program was designed by a multi-disciplinary team including physiotherapists, psychologists, occupational therapists, pediatricians, neonatologists and neonatal nurses, with the actual intervention to be carried out by a team comprising a physiotherapist and a psychologist.

Physiotherapy aims to improve functional use of movement and to limit disability[53] Postural control, that is the ability to control the body's position in space for stability and orientation, is not only important for development of gross and fine motor skills but may also be important for cognitive development. Movement enables infants to regulate their behavior (e.g. sucking thumb to self calm), interact with their family, other people and objects, and to respond to environmental demands[54]. The physiotherapist and the psychologist aim to improve the infant's postural control, behavioral regulation and mobility through education of parents on positioning, carrying and play ideas.

The psychological component of the program aims to support families on several levels. Firstly, supporting maternal mental health in the adjustment to mothering a preterm infant and discussing the environmental challenges that may be faced by the family when bringing home a preterm infant. Secondly, by providing an outlet for debriefing about the experience of preterm delivery and supporting the mother to deal with emotional reactions to preterm birth including guilt, loss, anger, sadness, anxiety and stress. In addition, standardized assessments will estimate clinical levels of depression using the EPDS at 6 months. The team will provide brief therapeutic intervention and referral for further support for symptoms of anxiety or depression, where indicated. Parents will be supported in creating social support networks to assist both physically and emotionally.

As a team the therapists will help parents to understand how their infant's physical and motor development/impairments are related to cognitive, social and emotional development. The therapists will explore concepts of positioning and stability to promote engagement with the environment and extend the concentration period, and they will discuss the use of social and emotional rewards as motivation for exploration (and thus movement). The importance of enriching the environment as the baby develops throughout the first year will be reinforced at each session, with appropriate information provided on developmental stages. This will be particularly emphasized through the importance of play, as it is through play that motor, cognitive, behavioral and language development occur.

The team will also support families with important issues, such as sleeping or feeding. Families will be encouraged to seek the support of their maternal child health nurses, doctor and other health professionals when the needs of the baby and family are beyond the scope of the intervention program.

#### Study Personnel

Intervention will be delivered by two study teams comprising an experienced pediatric physiotherapist and an experienced clinical psychologist. All four personnel will be trained prior to study commencement on the Brazelton Neonatal Behavioral Scale [55], and will spend time synchronising the content of the developmental modules.

#### Structure and content of program

The intervention involves the simultaneous processes of assessment and treatment. Each session involves the team visiting the family (infant and mother ± father or other primary caregiver) for 1.5-2.0 hours per session. During these sessions two key issues will be identified as concerns for the caregiver/s and concerns for the therapist. The session will be guided by the identified issues and subsequently interventions will be applied as the session progresses. In addition, these issues will serve as the basis for assessment in subsequent sessions for identification of ongoing concerns and will be used as a baseline to show developmental progress or change. The intervention program also has a structured content which is summarized in Table 1 and described in more detail.

**Table 1:** VIBeS Plus intervention program

Session	Age	Aims	Goals	Handouts
**1** **2**	1-2 weeks 1 month	1. To support parental mental health in the adjustment to parenting a preterm baby. 2. To assist the parents in understanding their baby's needs and abilities, and in responding in a sensitive and appropriate manner.	1. The opportunity for parents to talk about their journey into parenthood, the NICU experience and transition home. 2. To develop an understanding of how the surrounding environment has an affect the baby's development. 3. To recognize the baby's different states of arousal, cues and the baby's self-regulatory behaviors. 4. To assist the baby in reaching an organized state.	1. Reading your baby 2. Swaddling 3. States of arousal 4. Your social support team 5. Taking care of yourself

**3**	2 months	1. To increase the parent's confidence in understanding their baby's patterns, emerging skills and knowing how to respond appropriately to their developmental changes. 2. To establish routines for sleep, feed and play times. 3. To support parental mental health in the adjustment to having a preterm baby.	1. To understand the baby's patterns and their emerging skills. 2. For the parent to be aware of their baby's state and how this affects their ability to socialize and play in face-to-face interactions. 3. To demonstrate how positioning affects play and how the baby's movements are also important in the baby's social behavior.	1. Tummy Time 2. Understanding your baby

**4**	3 months	1. To explore parental behaviors that enhance parent-baby interaction. 2. To provide parents with both practical and emotional support.	1. For parents to recognize the relationship between parent and infant behaviors and its influence on interaction. 2. For parents to recognize the importance of different positions for play to encourage motor and social development.	1. Sitting 2. Lifting and Carrying

**5**	4 months	1. To assist the parents in understanding their baby's motor development and the importance of play in development. 2. To be aware of issues related to maternal mental health.	1. For parents to understand their baby's strengths and areas for improvement in relation to motor development. 2. To develop an understanding of how play affects all aspects of the baby's development including motor, cognitive and emotional development. 3. For parents to recognize times of stress and understand strategies to reduce stress.	1. Playing with your baby 2. Recognizing times of stress

**6**	6 months	1. For the parent to gain an understanding of their baby's developmental progress between ages 5-8 months. 2. To highlight the importance of different positions for development. 3. To assist parents with feeding transitions. 4. To promote parental well being.	1. To assist parents to develop strategies to promote their baby's development in motor, language, cognitive, social and emotional domains. 2. To provide the opportunity to discuss the transition of feeding: the introduction of solids, the changes in routines and any specific feeding issues. 3. Assessment of parental mental health with particular relevance to depression.	1. More Ideas for Sitting 2. Watching your baby grow (5-8 months) 3. Promoting your baby's development (5-8 months)

**7**	8 months	1. For parents to gain knowledge of their infant's current motor performance and strategies to enhance mobility. 2. For parents to understand their role in supporting their infant's independence.	1. For parents to understand their baby's strengths and areas for improvement in relation to motor development. 2. To create an enriched environment for the infant to develop, including space for mobility and age appropriate toys.	1. Watching your baby grow (9-12 months) 2. Promoting your baby's development (9-12 months)

**8**	9 months	1. For parents to recognize their baby as an independent person with their own temperament style. 2. For parents to gain an understanding of their role in encouraging their baby's independence through play.	1. To assist parents to read their child's behavioral cues and support their developing skills through participation in a structured play task. 2. To promote understanding of child's temperament and enhance parenting.	1. Encouraging Language Development 2. Parenting strategies

**9**	11 months	1. To prepare parents for the next 12 months of infant development in relation to motor, cognitive, language and social development. 2. To ensure parents feel supported after the intervention program is finished.	1. To discuss developmental progress to date and what is to be expected in the next 12 months. 2. For parents to understand how to deal with challenging behaviors as the infant becomes more independent and mobile. 3. To recommend ongoing referral to appropriate intervention services if required.	1. Development over the next 12 months 2. Dealing with challenging behavior

The sessions will be carried out in the family home, as home visits allow the family to be seen in their natural environment. This also allows for the caregiver/s to feed the infant and maintain their infant's regular sleep routine. When the sessions cannot be carried out at home, due to the infant being hospitalized or because caregivers work closer to the hospital, then a room in the outpatient facility at the Royal Women's Hospital or the Royal Children's Hospital will be used.

At the beginning of the intervention program parents will be given a folder to keep handouts that are given out at each session. The folder contains contact details of the team, an outline of the scheduled visits, a sheet to write down any questions to ask at the next visit and a record sheet of visits to other health professionals and concurrent interventions. Each session will include one to four handouts which summarize key points discussed during the session (Table 1). The handouts are designed to target key issues at different ages, such as tummy time at 2 months and promoting development from 5-8 months.

Each session will follow a structured format, which can be adapted for the infants' and caregivers' needs. The structure of the session will be dependent on the infant's state, so that if the infant is awake, then the intervention can begin with the focus on the infant. If the infant is asleep or being fed then the intervention can focus on the caregiver. At the beginning of each session the therapists will note any "arrival issues", such as other siblings being present, and "health issues", such as a recent admission to hospital or respiratory infection. This will be followed by the "Mother and Baby Scales" (MABS), which is a scale that assesses the baby's overall behavior and parental wellbeing[55] The MABS will be administered each session and a summary given to the parents during the final session to demonstrate the parents' and child's journey over the first 12 months. During each session there will also be two key questions asked based upon the Dolby et al[6] study:

"*What is the most positive thing about your baby?"*

"*What is the most challenging thing about your baby*?"

In addition, questions on sleeping and feeding will be asked and based upon these answers and general discussion, two key issues will be identified for the parent and therapist. These issues form part of the individualized intervention strategies, along with the generic intervention content listed in Table 1. In addition to the generic handouts mentioned previously, caregiver/s will be given an individualized strategy sheet at the end of the session, which has a list of no more than four practical strategies that address the key issues identified by the caregiver/s and therapists during the session. The strategies are designed to meet the needs of the caregiver/s and infant and consist of activities to encourage the infant's development, enhance parenting and/or well being. Specific developmental modules have been created to target these individual needs prior to commencement of the study. The modules are listed in Table 2.

**Table 2:** Developmental modules of VIBeS Plus program

Target area	
Sleeping	Mother's mental health
Feeding	Social support
States of arousal	Social interaction and play
Assisting baby's regulation and organization	Equipment and toys
Swaddling	Parenting to different temperaments
Positioning in the early months (0-6 months)	Separation and attachment
Positioning in the later months (6-11 months)	Enriching the movement
Carrying and lifting (0-3 months)	Developmental progress
Carrying and lifting (4-11 months)	Sensory stimulation
Upper limb reach (0-6 months)	Language development
Upper limb reach (6-12 months)	Parenting strategies
Mobility and transition movements	Recognizing times of stress

At the beginning of the next session parents will be asked whether each of the strategies was implemented and how successful it was on a visual analogue scale of 1 to 7 (1 = not at all, to 7=very). This information will be documented on the data collection sheets. Strategies will then be designed for the next session based upon the success of prior strategies and current key issues.

The data recording sheets will also be used to document the following: who attended the session, the duration and location of the session, the parent's and therapist's key issues, prioritise developmental concerns, rate success of any developmental modules that have been implemented, and the success rate of the intervention. The success of the developmental modules and intervention will be scored on the same visual analogue scale of 1 to 7. The success of the intervention will be rated for rapport, parental emotional availability, child's response to intervention and overall success of the intervention. These four areas will be given a subjective score after joint discussion between the physiotherapist and psychologist at the end of the session.

### Outcome Measures

All infants will be assessed by an examiner who is masked to group allocation. A flow diagram of the outcome measures is outlined in Figure 1.

The primary outcome measure is cognitive, language and motor development at two years' corrected age measured using the composite scores from the Bayley Scales of Infant and Toddler Development - 3^rd ^edition (Bayley-III) at 24 months' corrected age[41]. The Bayley-III is a norm referenced developmental scale of cognitive, language and motor development over the first 48 months that has good psychometric properties, and has been used extensively in follow-up of preterm infants.

The secondary outcome measures are as follows

*1. Motor Development *- At 12 and 24 months' corrected age the Neurological Sensory Motor Development Scale (NSMDA)[56] will be used to assess motor development. The NSMDA is a criterion-referenced test of gross and fine motor performance, neurological status, posture, balance and response to sensory input longitudinally. The Alberta Infant Motor scale (AIMs) [57] will be used to assess motor development at 12 months. The AIMS is a norm referenced observational motor assessment used for monitoring the gross motor development of typically-developing infants from birth up to 18 months. Both of these assessments have been selected as they have good psychometric properties, are geared to assessment of motor outcome of infants, are quick and easy to administer with minimal handling, and have relevance to the education of parents about their child's motor maturation.

*2. Infant Behavioral Regulation *- The Infant Toddler Social and Emotional Assessment (ITSEA) [58] will be used at 12 and 24 months. It is a comprehensive adult report measure of social -emotional problems and competencies in 1 to 3 year olds. It consists of 4 broad domains of behavior: dysregulation, externalizing, internalizing and competencies. Parents rate behaviors on a 3 point scale (0 = rarely/not true, 1 = somewhat true/sometime and 3 = Very true/often). It has good internal consistency for the 4 domains, good validity and test-retest reliability.

*3. Primary caregiver mental health *- will be measured with the Hospital Anxiety and Depression Scale (HADS)[59] which has 14 items in total (7 anxiety, 7 depression) at 24 months. Each item is scored with a four-point rating scale scored from 0 to 3, (0 = not at all, 3 = most) giving total scores ranging from 0 to 21 for each subscale and from 0 to 42 for overall distress. A score of 8-10 is suggestive of the presence of the affective state, and scores between 0-7 are within the normal range. It has been validated in a variety of settings and has been found to perform well in assessing the severity of anxiety disorders and depression, not only in primary care patients but also in the general population[60].

*4. Parenting *- The Parenting Stress Index Short Form (PSI-SF)[61] consists of a subset of 36 items drawn from the full version (PSI) organized into three, 12-item subscales: 1) Difficult Child, 2) Parent-Child Dysfunctional Interaction, and 3) Parent Distress and will be used at 24 months. The statements range from 'Strongly Agree' to 'Strongly Disagree'. The questionnaire provides a total stress score and 3 subscale scores, with lower scores indicating less stress. The Short-Form PSI has a correlation of 0.95 with the full length version[61].

*5. Family Burden *- will be assessed using two measures at both 12 and 24 months. The first is the Impact of Family Scale version G (IOF-G)[48] mentioned previously in the methods. The second measure is the Family Assessment Device (FAD) which consists of 60 items rated with a 4-point likert response format (strongly agree to strongly disagree) that assesses six dimensions of family functioning: Problem solving, Communication, Roles, Affective responsiveness, Affective involvement and Behavior control. Additionally, a General Functioning Scale assesses overall health pathology in the family. Both measures have demonstrated satisfactory reliability and validity[62].

*6. Social Support *- the Social Support Questionnaire (SSQ)[63] will be used at 24 months. It contains six items, and each item asks participants to: (a) list the people to whom they can turn and on whom they can rely in given sets of circumstances, and (b) indicate their level of satisfaction with these social supports. An individual's score represents the total level of satisfaction with their social support.

*7. Child-parent interaction *- The structured parent-child play interaction task[64] is an observational task which will be used to assess the quality of parent-child interactions and synchrony at 24 months. It was developed for use with very preterm infants and pre-schoolers, and is an adapted measure from the earlier work by the Dunedin Multidisciplinary Health and Development Research Unit,[65] the National Institute of Child Health and Human Development Study of Early Child Care and Chase-Lansdale et al[66]. The observational instrument consists of four different age-appropriate, problem-solving tasks. The tasks are designed to be challenging for the child and are administered in a set sequence based on the first 3 items' progressive degree of difficulty.

An independent rater will code both the parent and child's behavior for each problem solving task using a 5-point Likert scale. Parent behavior will be assessed using a coding scheme that includes: Positive affect (the overall quality of parent's positive expressions towards their child during each task); Negative affect (the intensity and frequency of the parent's degree of disapproval, anger, and negativism expressed toward the child while working on each task); Supportive presence (sensitivity, warmth and responsiveness to the child as they progress through each task); Facilitates self-regulation (instruction and support provided to the child to assist with successful task completion); and Intrusive/over controlling (Extent to which parent behavior is ill timed, intrusive, and excessively and inappropriately controlling relative to what the child is doing).

The coding scheme used to assess child behavior will include: Positive affect (the overall quality of positive expression/responses of the child during the task); Negative affect (the intensity and frequency of the child's degree of unhappiness, sadness, and hurt expressed during the task); Activity level (how motorically active the child is during each task); Child persistence (extent to which the child is actually problem-oriented on the task); Dependence (extent to which the child displays personal initiative in the situation or, conversely, expects the mother to provide direction and help); and Quality of task transitions (the ability of the child to move from one task to the next).

In addition to the parent and child codes, the dyads' 'interactional synchrony' will be coded on a 5-point Likert scale for each problem solving task. Interactional synchrony assesses the harmony, interconnectedness, responsiveness, reciprocity, engagement, mutual focus and shared affect of the dyad.

*8. Sensory Processing *- The Infant Toddler Sensory Profile Questionnaire[67] which is a parent report measure will be used to evaluate and identify patterns of sensory processing in six sensory systems including: General, Auditory, Visual, Tactile, Vestibular, and Oral Sensory Processing. It is suitable for use with 7 to 36 month olds and will be administered at 12 and 24 months' corrected age. Specific patterns of performance on the Infant/Toddler Sensory Profile Questionnaire have been shown to be indicative of difficulties with sensory processing and performance[67].

### Data Storage

Data will be recorded on paper files and entered electronically into a computerized database and stored securely. A combined database will be created for analysis by merging data from the perinatal and qualitative database, intervention database and outcome assessment data base using the statistical package Stata 10.0. Data will be checked and cleaned again using Stata following merging of files.

### Analysis plan

Analysis will follow standard principles for randomized controlled trials, using simple two-group comparisons performed using all subjects for whom outcome data are available, on an intention-to-treat basis. The primary comparison at 24 months will be based on the Bayley cognitive and motor composite scores, which will be compared between groups using linear regression, controlling for multiple births. Multivariate regression will be used to adjust for potential imbalances in major baseline confounders such as the extent of brain injury (and sociodemographic variables). Secondary analyses will use similar methods to compare the outcomes between groups for the additional motor (NSMDA, AIMs); behavioral (ITSEA), primary caregiver mental health and parent -child interactions measures (VIBES, EAS) at 24 months. For dichotomous outcomes, comparisons will be by chi-squared tests and where continuous data exhibit substantial skewness, non-parametric (Mann-Whitney U test) methods will be used for simple comparisons, and regression analyses performed after appropriate transformation of the outcome.

## Discussion

This paper outlines a preventative care program designed for preterm infants over the first year of life to improve motor, cognitive and behavioral development. The program is based upon theoretical models and includes components of previously successful interventions. It is designed to be feasible to implement in a community setting, and to be able to be generalized across different groups in Australia and internationally. This study provides a unique opportunity to determine if a preventative care program will improve motor, cognitive, and behavioral outcomes at two years of age, as well as caregiver mental health.

## Abbreviations

EPDS: Edinburgh Postnatal Depression Scale; CISS: Coping Inventory for Stressful Situations; IOF-G: Impact of Family Scale; STAI: State Trait Anxiety Inventory; PSI: Parenting Stress Index; WMI: white matter injury; BSITD-III: Bayley Scales of Infant and Toddler Development -3^rd ^edition; AIMS: Alberta Infant Motor Scale; NSMDA: Neurological Sensory Motor Developmental Assessment; ITSEA: Infant Toddler Social Emotional Assessment; HADS: Hospital Anxiety and Depression Scale; PSI-SF: Parenting Stress Index - Short Form; SSQ: Social Support Questionnaire; FAD: Family Assessment Device.

## Competing interests

The authors declare that they have no competing interests.

## Authors' contributions

LD, TI, RB are chief investigators for this study and were involved in the conception of the study. AS, CF, PA, JO, RB, TE, LD designed the study and intervention package. AS, CF, JO, LB were involved in data collection and delivering the intervention program. AS drafted the manuscript with all authors providing critical review and final approval. AE will be involved in the independent assessments at 2 years of age. PA, RB, TE and LD are involved with supervision of students on this project.

## Pre-publication history

The pre-publication history for this paper can be accessed here:

http://www.biomedcentral.com/1471-2431/9/73/prepub
